# Estimating global and regional morbidity from acute bacterial meningitis in children: assessment of the evidence

**DOI:** 10.3325/cmj.2013.54.510

**Published:** 2013-12

**Authors:** Ivana Lukšić, Rosanda Mulić, Rachel Falconer, Mirjana Orban, Simrita Sidhu, Igor Rudan

**Affiliations:** 1 Department of Microbiology, Institute of Public Health “Dr Andrija Štampar,” Zagreb, Croatia; 2 Department of Public Health, Faculty of Medicine, University of Split, Split, Croatia; 3 Centre for Population Health Sciences, University of Edinburgh, Medical School, Edinburgh, Scotland, UK; 4 Department of Mental Health and Drug Prevention, Institute of Public Health “Dr Andrija Štampar,” Zagreb, Croatia

## Abstract

**Aim:**

To estimate global morbidity from acute bacterial meningitis in children.

**Methods:**

We conducted a systematic review of the PubMed and Scopus databases to identify both community-based and hospital registry-based studies that could be useful in estimation of the global morbidity from bacterial meningitis in children. We were primarily interested in the availability and quality of the information on incidence rates and case-fatality rates. We assessed the impact of the year of study, study design, study setting, the duration of study, and sample size on reported incidence values, and also any association between incidence and case-fatality rate. We also categorized the studies by 6 World Health Organization regions and analyzed the plausibility of estimates derived from the current evidence using median and inter-quartile range of the available reports in each region.

**Results:**

We found 71 studies that met the inclusion criteria. The only two significant associations between the reported incidence and studied covariates were the negative correlation between the incidence and sample size (*P* < 0.001) and positive correlation between incidence and case-fatality rate (*P* < 0.001). The median incidence per 100 000 child-years was highest in the African region – 143.6 (interquartile range [IQR] 115.6-174.6), followed by Western Pacific region with 42.9 (12.4-83.4), the Eastern Mediterranean region with 34.3 (9.9-42.0), South East Asia with 26.8 (21.0-60.3), Europe with 20.8 (16.2-29.7), and American region with 16.6 (10.3-33.7). The median case-fatality rate was also highest in the African region (31.3%). Globally, the median incidence for all 71 studies was 34.0 (16.0-88.0) per 100 000 child-years, with a median case-fatality rate of 14.4% (5.3%-26.2%).

**Conclusions:**

Our study showed that there was now sufficient evidence to generate improved and internally consistent estimates of the global burden of acute bacterial meningitis in children. Although some of our region-specific estimates are very uncertain due to scarcity of data from the corresponding regions, the estimates of morbidity and case-fatality from childhood bacterial meningitis derived from this study are consistent with mortality estimates derived from multi-cause mortality studies. Both lines of evidence imply that bacterial meningitis is a cause of 2% of all child deaths.

Meningitis is an infectious disease affecting the brain membrane and spinal cord (1). Globally, bacterial meningitis is the most severe type of meningitis, mainly caused by a triad of species *Neisseria meningitidis, Streptocccus pneumonia,* and *Haemophilus influenzae* (2). While viral meningitis is usually a self-limiting disease with good prognosis, bacterial meningitis is potentially fatal, requiring urgent medical assistance and management with antibiotics treatment (3). Various estimates of the burden of bacterial meningitis have been proposed to date, but they have mainly focused on mortality (4), long-term sequels (5), or etiology-specific morbidity and mortality (6-8).

Interestingly, there have been no comprehensive attempts to estimate the overall global burden of bacterial meningitis in children. This is not surprising, because such attempt would face almost insurmountable methodological challenges. First, there is a problem with case definition of “bacterial meningitis” (9). In low resource settings, where the problem is most common, many children may present with “purulent meningitis,” whose cause is highly likely bacterial, but laboratory capacity may not be sufficient to isolate the causal agent and confirm the diagnosis. This leads to a discrepancy between morbidity burden estimates based on “all purulent meningitis” and “laboratory confirmed meningitis” – the latter always being lower than the former, but to a different extent in different contexts (10). The second large methodological obstacle is the problem of “meningitis belt.” The meningitis belt is the band of countries extending from Senegal to Ethiopia, characterized by semi-arid climate, dry seasons, and dusty winds, with seasonal outbreaks of meningococcal meningitis being recorded since the beginning of the 20th century (11). The problem with these epidemics is that they can last for several years and dramatically change the importance of meningococcus in comparison to the other two bacterial agents (*S. pneumoniae* and *H. influenzae*) both regionally and globally (11). This makes it difficult to express the “burden of disease” for any given year, because it will be very different in intra-epidemic and inter-epidemic years. Moreover, the extent of vaccine coverage against *N. meningitidis*, *S. pneumoniae* and *H. influenzae* is changing the burden rapidly and rather dramatically in many places, rendering the scarce evidence from before the period of vaccination rather useless and indicates a need of revision (12). Finally, the emergence of HIV/AIDS pandemic led to a substantial number of infected children, whose resistance to other infections is impaired and they present a specific category of population in which the rates of incidence and case-fatality rates may be very different from those in other children (13).

It is apparent that meningitis continues to contribute significantly to global mortality and morbidity, but the impact of the efforts to control it is difficult to estimate given that we do not have comprehensive estimates of global morbidity patterns. Understanding the global morbidity from bacterial meningitis would be useful because it would also help to validate the existing mortality estimates through application of appropriate case-fatality rates. The purpose of the present study is to provide a comprehensive assessment of the evidence that is available for estimating the global morbidity from acute bacterial meningitis in children globally. We will also propose initial, robust estimates of the burden, with suggestions on the possible ways to address the methodological challenges in future studies.

## Methods

### Data sources – search strategy and selection criteria

It is essential to formulate a case definition for meningitis given that the clinical presentation is often non-specific (Supplementary Text 1)(supplementary text 1). Therefore, we only included studies that aimed to confirm suspected meningitis cases with the analysis of cerebrospinal fluid (CSF) (Supplementary Text 2) (supplementary text 2) to eliminate the potential for diagnostic subjectivity and allow comparison. In addition, ICD-10 codes were noted, with those applicable to meningitis used in making a decision on study inclusion (Supplementary Text 3)(supplementary text 3).

The search strategy was defined with the aim to capture all possibly informative studies, while excluding the majority of studies that were not relevant. No studies were excluded based on language of publication, although for studies not published in English information was extracted from abstracts only.

**Figure 1:**
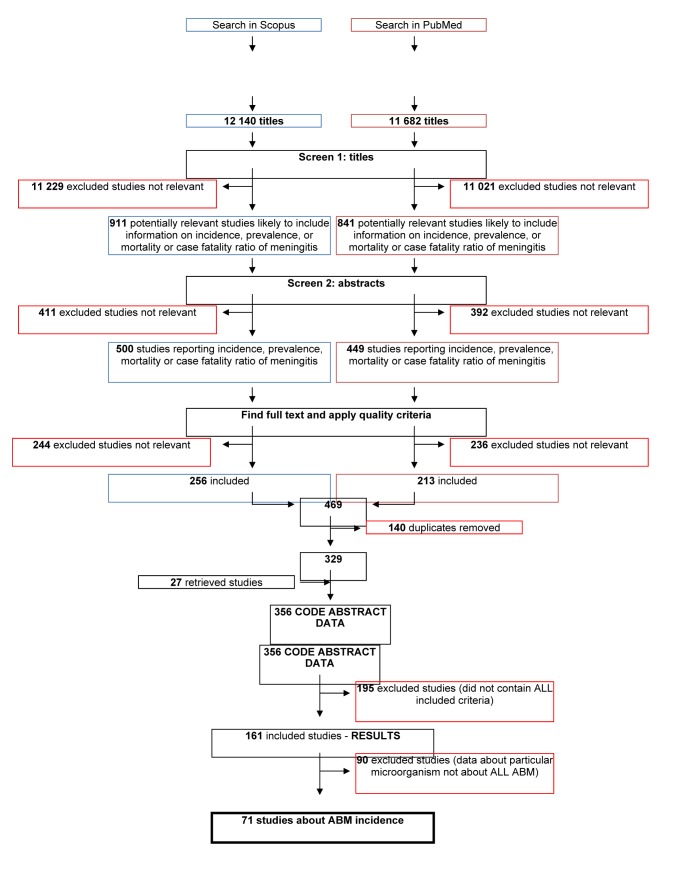
Flowchart showing the process of literature search. ABM – acute bacterial meningitis.

The search strategy used was:

“meningitis AND (incidence OR prevalence OR mortality OR morbidity OR sequela* OR case fatality OR risk factor*)”

and was applied as a free search in title/abstract field in both the PubMed and Scopus databases in the period between January 1, 1980 and December 31, 2010. The search was limited to studies in humans only (Figure 1).

The full text of the studies was retrieved wherever possible. The following were applied as inclusion criteria required for each study before proceeding to data extraction:

(i) Incidence, prevalence, mortality or case fatality rate for meningitis;

(ii) Defined denominator of the study population (community or population based studies were preferred, but hospital-based registries of sufficient quality and with a defined reference population were also retained);

(iii) Clinical confirmation of meningitis by CSF culture;

(iv) Minimum study period of one year (or multiples of one year);

(v) At least 10 cases of confirmed acute bacterial meningitis;

(vi) Age groups defined as 0-4 years, because in the studies that used age groups 0-2 years and/or 0-14 years, the overall incidence would be substantially different;

(vii) Any further methodological ambiguities about how the study was conducted, or systematic error in the design of research that could affect the final results of the study;

The data extracted from studies was entered into a database for further analysis (Supplementary Table 1)(supplementary table 1).

The titles returned by the first search were further browsed by two independent researchers (IL and RF), who selected the final set of studies that met all inclusion criteria. Wherever two researchers disagreed about inclusion or exclusion of certain studies, disagreement was resolved by seeking the opinion from a third party (IR) who resolved any disputes.

### Data arrangement

The incidence rate of meningitis in children was determined using the following formula:

I = (number of new cases ×100,000) / (population at risk × number of years of follow-up)

The resulting incidence was not age-standardized to the level of individual countries or regions, because of the lack of data on the level of individual studies or registries to enable direct age standardization using the age-specific incidence of five-year age groups, which was a prerequisite for the implementation of such standardization and which would represent the ideal design. However, in all studies incidence was expressed in a standard way, as the number of new cases per year per 100 000 person-years.

Case-fatality rate was expressed in all studies as a percentage, and its value was calculated as:

CFR = (number of deaths from meningitis) / (number of cases of meningitis)

### Statistical analysis

The association between the dependent variable (incidence rate) and its independent predictors was studied in the following way:

(i) A regression analysis was performed to analyze the relationship between the design of study (prospective or retrospective) and the reported incidence rates and the relationship between the study setting (community-based or hospital registry) and the reported incidence rates. In this way, we were primarily interested whether the lack of access to hospitals would affect the reports based on hospital registers.

A regression analysis based on linear, “power,” logarithmic, and/or exponential function was performed to analyze:

(ii) the relationship between the year of study and the reported incidence rates, to check for any apparent time trends. The results were shown as the “best fit” equation showing dependence of incidence rate on the year of the study and the value of R^2^ (eg, the proportion of the total variability in the dependent variable explained by the predictor variable).

(iii) the relationship between the duration of the study (in months) and incidence rates for acute bacterial meningitis. This analysis was primarily important to check for the possible presence of the “Hawthorne effect” – ie, indirect positive effects of carrying out the study in the population, leading to improved health indicators over time.

(iv) the relationship between sample size and the reported incidence rates. This analysis is important as it can detect “publication bias.” Generally, with the increasing sample sizes, the incidence rates should converge to the real value, while in smaller studies there is “scattering” in both directions. Another possible problem is that larger studies may report lower incidence rates because of difficulties in monitoring a very large population, which results in under-reporting.

(v) whether the incidence rate may itself serve as a predictor, with case-fatality rate as a dependent variable.

The statistical significance of the association was expressed as a *P* value and values lower than 0.01 were considered significant. All the analyses were conducted using Microsoft Excel software licensed to the University of Edinburgh.

## Results

Seventy-one retained studies met minimum requirements for inclusion (Table 1). Most studies were available from the African Region – 21 in total, 14 from Europe, 13 from Western Pacific region, 11 from the Americas, 7 from the Eastern Mediterranean region, and 5 from South East Asia. The median incidence was highest in the African region, where the “meningitis belt” is located – 143.6 new cases per 100 000 child-years (IQR 115.6-174.6), followed by Western Pacific region with 42.9 (12.4-83.4) and the Eastern Mediterranean region with 34.3 (9.9-42.0). The median case-fatality rate was also highest in the African region (31.3%). Globally, the median incidence rate for all 71 studies was 34.0 (16.0-88.0) of new cases per 100 000 child-years, with a median case-fatality rate of 14.4% (5.3%-26.2%).

**Table 1:** Medians of incidence and lethality rates of acute bacterial meningitis in children under 5 y of age by World Health Organization (WHO) regions and in the world. Presented are interquartile ranges and the minimum and maximum of observed values.

WHO region	Number of studies	Median incidence rate (per 100 000 child-years)	Interquartile range	Minimum	Maximum	Median case-fatality rate	Interquartile range	Minimum	Maximum
Africa	21	143.6	115.6-174.6	43.4	311.0	0.313	0.262-0.317	0.203	0.388
Americas	11	16.6	10.3-33.7	8.5	59.6	0.153	0.141-0.207	0.092	0.396
Eastern Mediterranean	7	34.3	9.9-42.0	3.1	43.0	0.063	0.052-0.105	0.041	0.146
Europe	14	20.8	16.2-29.7	8.9	36.6	0.081	0.054-0.107	0.053	0.200
South-East Asia	5	26.8	21.0-60.3	14.2	66.8	0.037	0.033-0.041	0.029	0.045
Western Pacific	13	42.9	12.4-83.4	5.3	149.6	0.078	0.048-0.117	0.033	0.184
World	71	34.0	16.0-88.0	3.1	311.0	0.144	0.053-0.262	0.029	0.396

**Figure 2:**
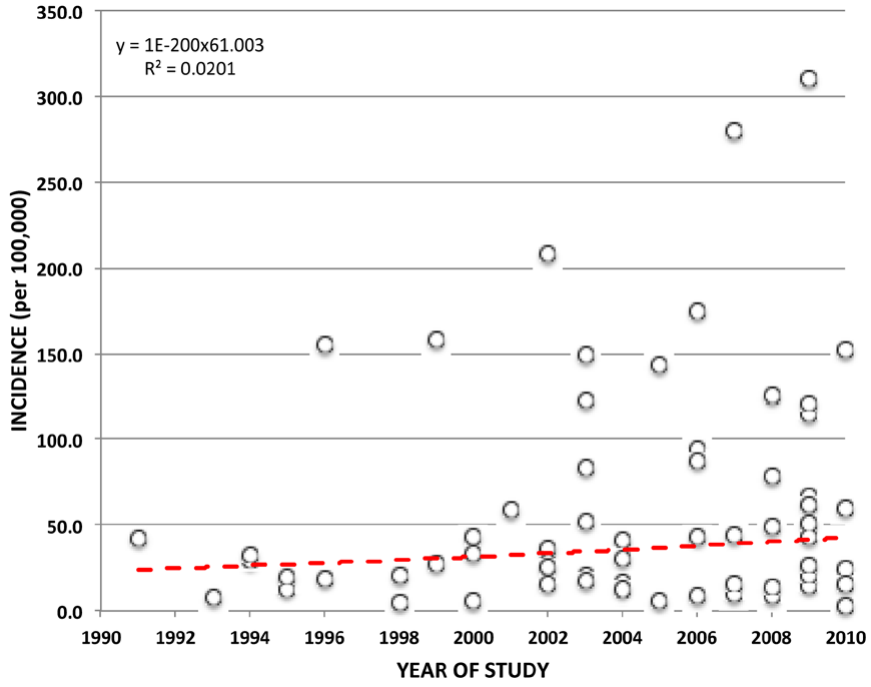
Analysis of the relationship between the year of study and the reported incidence rates for acute bacterial meningitis.

**Figure 3:**
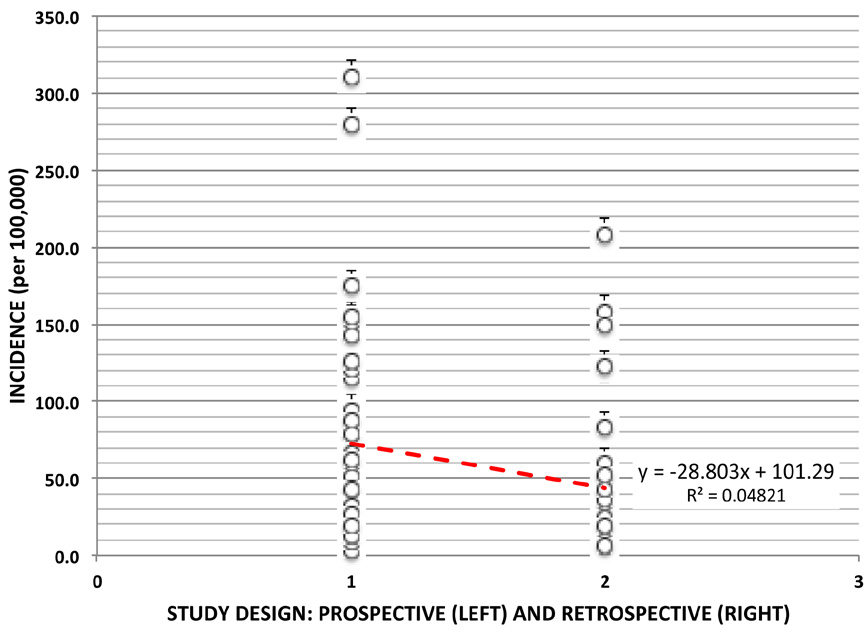
Analysis of the relationship between study design (prospective/retrospective study) and incidence rates for acute bacterial meningitis.

**Figure 4:**
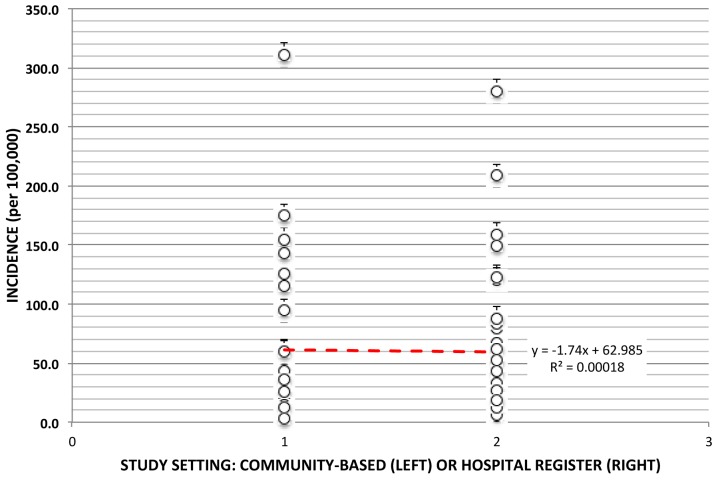
Analysis of the relationship between study setting (community based vs hospital registers) and incidence rates for acute bacterial meningitis.

**Figure 5:**
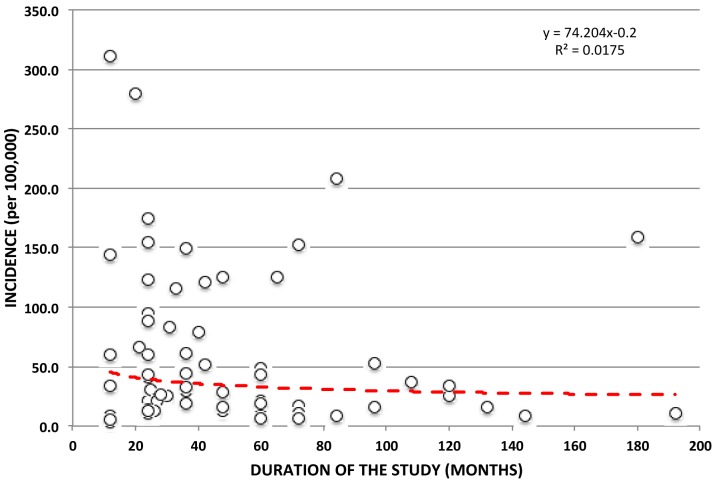
Analysis of the relationship between the duration of the study (expressed in months) and incidence rates for acute bacterial meningitis.

In order to further discuss various effects of obtained covariates on observed results we conducted several analyses of the association between the criterion variable (incidence rate) and the variables associated with the design of the research. In the first such test, the relationship between the study year and the reported incidence rates was explored for all types of confirmed acute bacterial meningitis (Figure 2), to investigate whether there was a growing or a declining trend in incidence over time. Although it appears that the incidence increased over the two decades from about 30 to about 40 new cases per 100 000 person-years, this approximation explains only about 2% of the observed variability in the system. The median incidence reported in prospective studies was about 75 cases per 100 000 child-years and in retrospective about 45 cases per 100 000 child-years (Figure 3), which indicates that retrospective studies generally report lower incidence than prospective studies, but this difference was not significant. The analysis of influence of study setting on the results did not show any systematic influence (Figure 4). Moreover, we analyzed the effect of the study duration on reported incidence rates (Figure 5). This analysis aims to detect the presence of the “Hawthorne's effect.” Our study did not show any support for the existence of such effect.

**Figure 6:**
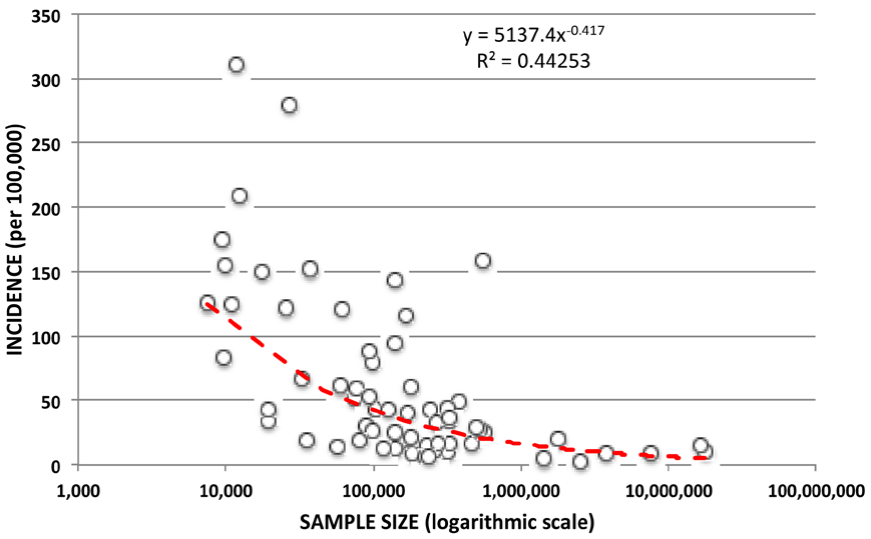
Analysis of the relationship between sample size and incidence rates for acute bacterial meningitis.

**Figure 7:**
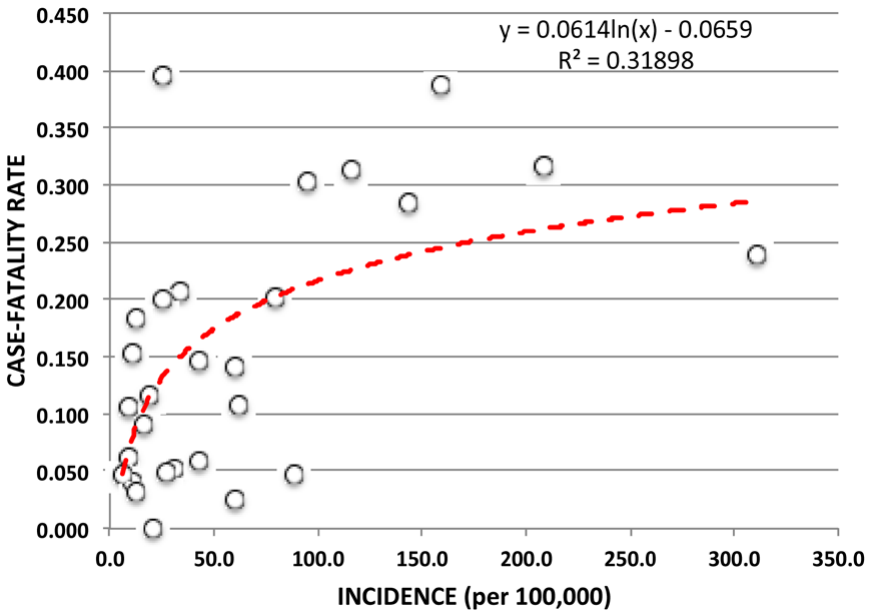
Analysis of the relationship between the incidence rates and the case fatality rates for acute bacterial meningitis.

We also explored the relationship between sample size and reported incidence (Figure 6). This analysis is very important, because it can demonstrate the existence of the “positive publication bias.” Generally, increased sample size leads to more credible results that begin to converge to the true value, while smaller studies can show greater or smaller estimates “scattered” in both directions around the true values, because there is an increased likelihood of false-positive results in smaller samples. In this study, there was a significant reduction in incidence with increasing sample size (with R^2^ of 44.3%, *P* < 0.0001). This finding could perhaps be partly explained by difficulties in systematic prospective follow-up of all patients in very large studies, resulting in decreased reporting of the cases and smaller incidence. Finally, when the dependent variable was changed, the incidence became a predictor variable, while the case-fatality rate became a new criterion variable – because time sequence between these two variables requires such approach (Figure 7). With the increase in the incidence rate, there was a strong rise in case-fatality rate, too, but only to the extent of about 30%, when the “saturation” occurs and lethality remains approximately the same thereafter, regardless of further increase in incidence.

## Discussion

This study provides new insights relevant to health policy development toward the fight against acute bacterial meningitis in preschool children. It assesses the available evidence in a systematic way to demonstrate that there is now a sufficient number of studies to allow a robust initial estimation of the global burden of meningitis. This is becoming possible due to a sufficient number of studies (71 papers) that implemented comparable methods and provided results that were consistent with each other.

In this study, we did not attempt to overcome the main obstacles to computing the burden of meningitis at the global level – the problem of etiologically unaccounted cases of purulent meningitis, multi-year epidemics in the meningitis belt, correction of the reported results for vaccination coverage, or accounting for the effect of HIV/AIDS positivity. The aim here was to simply present an initial, crude assessment of the burden based on the number of observed cases that were etiologically confirmed, in order to present a credible lower limit to the estimates of global and regional burden of childhood acute bacterial meningitis and explore whether it yielded plausible results that were compatible with previous estimates. Further adjustments and etiology-specific estimates are beyond the scope of this article, but we plan to continue our efforts and address them with increasing amount of appropriate evidence.

Accurate estimation of global, regional, and national burden meningitis in children will remain difficult for a number of reasons. The incidence of meningitis in the population generally can be adequately estimated only by longitudinal studies in the community. Such studies are rare in developing countries, where the burden of meningitis is the greatest, partly because such studies require a greater commitment of researchers and investors over a longer period of time. Due to the seasonal nature of meningitis frequency, which has different peaks of viral and bacterial outbreaks at different times, it is necessary to carry out research that would measure the frequency throughout the entire calendar year (or multiples of 12-month period). This problem is typical for many infectious diseases, such as infant diarrhea, pneumonia, or flu, but meningitis has additional specificity that is almost impossible to resolve methodologically, ie, that it can last for several years, for example in the “meningitis belt” in Africa. In these areas, a proper assessment of the epidemiological characteristics of the disease would require studies that would take a minimum of 10 years or more, so that a proper insight into the morbidity and mortality in the inter-epidemic and intra-epidemic periods could be obtained. This preliminary study that assessed the availability and quality of the current evidence and provided the first robust estimates of the morbidity from bacterial meningitis in children should therefore be amended in the future by adequate adjustments. Those adjustments would need to take into account the multi-year epidemics in the “meningitis belt” issue, HIV/AIDS positivity, and vaccine coverage.

In addition to those methodological requirements, a further source of uncertainty in measuring the frequency of meningitis in children in the community results from the case definition of meningitis, as well as the adequacy of the application of this definition by the assessors to make a diagnosis. There is no definition of clinical symptoms of the disease that is perfectly correct and distinguishes cases of meningitis from other possible diseases with 100% validity. Therefore, any study of the incidence of meningitis in the community would not measure the meningitis as a disease, but rather the number of children who test positive for the chosen definition of a case of meningitis. The definition of a case can be based on a number of clinical signs and symptoms, and diagnostic procedures, as well as on laboratory isolation of pathogens from cerebrospinal fluid. Depending on the combination of sensitivity and specificity of selected case definitions of meningitis, the burden of meningitis in the community can be over- or underestimated.

In this study, we separated all retrieved studies according to WHO regions: Africa (AFRO), the Americas (AMRO), Eastern Mediterranean region (EMRO), Europe (EURO), Southeast Asia (SEARO), and Western Pacific (WPRO). We calculated regional burden of morbidity and appropriate confidence intervals as the median of all standardized estimates (and inter-quartile range, IQR) multiplied by the size of the population of children aged 0-4 years in the year 2010. Estimates were based on 71 independent studies published between 1980 and 2010. Finally, we multiplied the burden calculated as above with median reported case-fatality rate for each region to estimate the total number of deaths and compare it to previous estimates, using UN's population projections for 2010 (14). Concordance with previous studies means that all the presently available evidence on the epidemiology of acute bacterial meningitis in children is beginning to converge toward a consistent picture on the extent and significance of this devastating disease in the world today. This justifies further efforts to refine etiology-specific estimates and work toward generating national-level estimates.

Moreover, it is important to note that there is a considerable discrepancy between the estimates based on the clinical picture of purulent meningitis and estimates based on laboratory-confirmed bacterial meningitis. The former are likely to overestimate the actual size of the problem because they may involve complicated cases of meningitis that was primarily viral in etiology. The latter probably underestimate the true magnitude of the problem because they fail to isolate and prove the cause in a proportion of all cases due to problems related to laboratory analysis and variability of pathogens in poorly developed regions, where the burden of disease is greatest. Our study implies that confirmed cases of bacterial meningitis account for about 61.5% of the burden of all purulent meningitis globally.

Regardless of the aforementioned weakness and uncertainties inherent to this preliminary study, the main strength of this study is that it used the two most accessible sources of data – PubMed and Scopus – to demonstrate that there is now sufficient evidence to generate improved and internally consistent estimates of the global burden of acute bacterial meningitis in children. We only used PubMed and Scopus because we assumed that those two sources would account for a large majority of studies that would satisfy our stringent inclusion criteria, and this study was of an exploratory character and we did not aim to be fully comprehensive at this stage of the analysis. Some of our region-specific estimates are still very uncertain due to scarcity of data from the corresponding regions, which may in some cases lead to rather implausible regional estimates (such as case-fatality rates in Americas, for example, which seem rather high in comparison to other, less developed regions). However, the main positive conclusion is that the estimates of morbidity and case-fatality from childhood bacterial meningitis derived from this study are consistent with mortality estimates derived from multi-cause mortality studies. Both lines of evidence imply that bacterial meningitis is a cause of 2% of all child deaths, which is *de facto* a replication of the result obtained through an entirely independent line of evidence. This gives us assurance that the global estimates of mortality from bacterial meningitis are plausible and that this new estimate of morbidity is rater robust, too.

According to this study, if case-fatality rates were applied to incidence rates, then the total number of deaths from meningitis in children younger than 5 years annually in the world would exceed 113 000 children, which is 1.64% of all child deaths in the world in 2010. This number is supportive of the estimates of Child Health Epidemiology Reference Group (CHERG), which amounted to 2% and were based on an entirely different set of evidence compared to our present study, using only information from multi-cause mortality studies (4). Thus, this study indicates that there is an increasing consistency between the current estimate of the global burden of morbidity from bacterial meningitis and the estimate of mortality.

This study provided the first robust estimate of the global and regional burden of acute bacterial meningitis. It also confirmed that the current estimate of the mortality from meningitis based on multi-cause mortality studies is likely to be accurate. The presented evidence should allow further and improved estimates, and also planning and guidelines for health-related activities for bacterial meningitis in preschool children globally and regionally, with the aim of combating morbidity and mortality from this disease. It is clear that the increase in pneumococcal, Hib, and meningococcal vaccination, which are currently carried out by the GAVI Alliance and UNICEF (15,16) will contribute to a further significant reduction of morbidity and mortality from bacterial meningitis in children all over the world.
