# Supplementary material for: Estimating global and regional morbidity from acute bacterial meningitis in children: assessment of the evidence
Source: Croat Med J. 2013 Dec;54(6):510–8. doi: 10.3325/cmj.2013.54.510 (PMC3893986; doi:10.3325/cmj.2013.54.510)
Supplement: Supplementary Text 1 [file CroatMedJ_54_s012.pdf]

Supplementary text 1: Meningitis definition

Acute bacterial disease with sudden onset and fever, intense headache, nausea, vomiting, neck stiffness and – in meningococcal disease – petechial rash with pink macules. The disease must be accompanied by laboratory evidence (in cerebrospinal fluid or blood) of *Neisseria meningitidis, Streptococcus pneumoniae* or *Haemophilus influenzae type b*.
Streptococcus pneumoniae – Acute bacterial disease with sudden onset and fever, intense headache, nausea, vomiting, and neck stiffness. The disease must be accompanied by laboratory evidence (in cerebrospinal fluid or blood) of *S. pneumoniae.*
Haemophilus influenzae – Acute bacterial disease with sudden onset and fever, intense headache, nausea, vomiting, and neck stiffness. The disease must be accompanied by laboratory evidence (in cerebrospinal fluid or blood) of *H.influenza* type B.
Neisseria meningitidis – Acute bacterial disease with sudden onset and fever, intense headache, nausea, vomiting, and neck stiffness. The disease must be accompanied by laboratory evidence (in cerebrospinal fluid or blood) of *N. meningitidis.*
Meningococcaemia without meningitis - Invasion of the bloodstream with *N. meningitidis*.
