# Supplementary material for: Estimating global and regional morbidity from acute bacterial meningitis in children: assessment of the evidence
Source: Croat Med J. 2013 Dec;54(6):510–8. doi: 10.3325/cmj.2013.54.510 (PMC3893986; doi:10.3325/cmj.2013.54.510)
Supplement: Supplementary Text 1 [file CroatMedJ_54_s013.pdf]

Supplementary text 2: Case definition based on cerebrospinal fluid indices for meningitis (Boon et al 2004)

Viral meningitis:<br><br>Lymphocytes 10 – 2000 mm^3<br>Normal glucose and protein<br>Negative Gram stain
Bacterial meningitis:<br><br>Polymorphs 1000 – 5000 mm^3<br>Low glucose and normal/elevated protein<br>Positive Gram stain
Tuberculous:<br><br>Polymorphs/lymphocytes/mixed 50 – 5000 mm^3<br>Low glucose and elevated protein<br>Gram stain may be negative
Fungal:<br><br>Lymphocytes 50 – 500 mm^3<br>Low glucose and elevated protein<br>Gram stain may be positive or negative
Malignant:<br><br>Lymphocytes 0 – 100 mm^3<br>Low glucose and normal or elevated protein<br>Negative Gram stain
**Clinical presentation** – combination of pyrexia, headache, neck stiffness. The severity of symptoms varies with the causative organism, as does the presence of other features – for example, skin rash.
**Gold standard case definition – cases confirmed by CSF culture from lumbar puncture** showing combination of features consistent with particular cause including elevated cell count and alternations in glucose/protein levels.
