# Supplementary material for: Estimating global and regional morbidity from acute bacterial meningitis in children: assessment of the evidence
Source: Croat Med J. 2013 Dec;54(6):510–8. doi: 10.3325/cmj.2013.54.510 (PMC3893986; doi:10.3325/cmj.2013.54.510)
Supplement: Supplementary Text 3 [file CroatMedJ_54_s014.pdf]

Supplementary text 3 - ICD-10 Codes

**Inflammatory diseases of the central nervous system (G00-G09)**
**G00 Bacterial meningitis, not elsewhere classified**
Includes :
· arachnoiditis<br>· leptomeningitis<br>· meningitis<br>· pachymeningitis
Excludes:
bacterial:<br>· meningoencephalitis (G04.2)<br>· meningomyelitis (G04.2)
**G00.0 Haemophilus meningitis**
**Meningitis due to Haemophilus influenzae**
**G00.1 Pneumococcal meningitis**
**G00.2 Streptococcal meningitis**
**G00.3 Staphylococcal meningitis**
**G00.8 Other bacterial meningitis**
Meningitis due to:
· Escherichia coli<br>· Friedländer bacillus<br>· Klebsiella
**G00.9 Bacterial meningitis, unspecified**
Meningitis:<br>· purulent NOS<br>· pyogenic NOS<br>· suppurative NOS
**G01* Meningitis in bacterial diseases classified elsewhere**
Meningitis (in):<br>· anthrax (A22.8+)<br>· gonococcal (A54.8+)<br>· leptospirosis (A27.-+)<br>· listerial (A32.1+)<br>· Lyme disease (A69.2+)<br>· meningococcal (A39.0+)<br>· neurosyphilis (A52.1+)<br>· salmonella infection (A02.2+)<br>syphilis:<br>· congenital (A50.4+)<br>· secondary (A51.4+)<br>· tuberculous (A17.0+)<br>· typhoid fever (A01.0+)
Excludes: meningoencephalitis and meningomyelitis in bacterial diseases classified elsewhere (G05.0*)
**G02* Meningitis in other infectious and parasitic diseases classified elsewhere**
**Excludes:** meningoencephalitis and meningomyelitis in other infectious and parasitic diseases classified elsewhere (G05.1-G05.2*)
**G02.0* Meningitis in viral diseases classified elsewhere**

Meningitis (due to):<br>· adenoviral (A87.1+)<br>· enteroviral (A87.0+)<br>· herpesviral [herpes simplex] (B00.3+)<br>· infectious mononucleosis (B27.-+)<br>· measles (B05.1+)<br>· mumps (B26.1+)<br>· rubella (B06.0+)<br>· varicella [chickenpox] (B01.0+)<br>· zoster (B.02.1+)
**G02.1* Meningitis in mycoses**
Meningitis (in):<br>· candidal (B37.5+)<br>· coccidioidomycosis (B38.4+)<br>· cryptococcal (B45.1+)
**G02.8* Meningitis in other specified infectious and parasitic diseases classified elsewhere**
Meningitis due to:<br>· African trypanosomiasis (B56.-+)<br>· Chagas' disease (chronic) (B57.4+)
**G03 Meningitis due to other and unspecified causes**
Includes:
· arachnoiditis<br>· leptomeningitis<br>· meningitis<br>· pachymeningitis
Excludes:
· meningoencephalities (G04.-)
· meningomyelitis (G04.-)
**G03.0 Nonpyogenic meningitis**
Non bacterial meningitis
**G03.1 Chronic meningitis**
**G03.2 Benign recurrent meningitis [Mollaret]**
**G03.8 Meningitis due to other specified causes**
**G03.9 Meningitis, unspecified**
Arachnoiditis (spinal) NOS
**G09 Sequelae of inflammatory diseases of the central nervous system**
Note: This category is to be used to indicate conditions whose primary classification is to G00-G08 (i.e. excluding those marked with an asterisk (*)) as the cause of sequelae, themselves classifiable elsewhere. The "sequelae" include conditions specified as such or as late effects, or those present one year or more after onset of the causal condition. For use of this category reference should be made to the relevant morbidity and mortality coding rules and guidelines in Volume 2.
Source: World Health Organization, 2007 10th Revision
Available from: http://www.who.int/classifications/apps/icd/icd10online/
