# Supplementary material for: Estimating global and regional morbidity from acute bacterial meningitis in children: assessment of the evidence
Source: Croat Med J. 2013 Dec;54(6):510–8. doi: 10.3325/cmj.2013.54.510 (PMC3893986; doi:10.3325/cmj.2013.54.510)
Supplement: Supplementary Table 1 [file CroatMedJ_54_s015.pdf]

Supplementary Table 1. The most important characteristics of a total of 71 studies on the incidence of acute bacterial meningitis that met the minimum requirements necessary for inclusion in the study of the global burden of acute bacterial meningitis in children; in the column "study design" "1" means a prospective and "2" retrospective studies; considering the setting of the study, "1" means a study conducted in a defined community-based sample and "2" studies based on hospital or other registers of patients, while shortcuts for diagnostic criteria indicate the following tests: G-Gramm, BC-bacterial culture; LA-latex agglutination, CC-cell count, PCR-polymerase chain reaction; CSF-isolation from the cerebrospinal fluid, B-isolation from the blood; S-serology, NP-not presented.

AUTHOR/YEAR OF PUBLICATION	WHO REGION	STUDY SITE	STUDY DESIGN	STUDY DURATION (mo)	STUDY SETTING	DIAGNOSTIC CRITERIA (see legend for abbreviations)	NUMBER OF CASES (CONFIRMED ALL; PRO YEAR)	POPULA-TION DENOMI-NATOR	INCIDENCE (per 100 000, AGE <5 y)	MORTALITY (per 100 000, AGE <5 y)
Kisakye et al., 2009	AFR	Kampala, Uganda	1	60	2	CC, CSF, LA	529	243774	43.4	NP
Lewis et al., 2008	AFR	Kampala, Mbarara, Gulu, Uganda	1	60	2	CC, CSF	925	379075	48.9	NP
Kisakye et al., 2009	AFR	Mbarara, Uganda	1	42	2	CC, CSF	133	74103	51.3	NP
Iriso et al., 2008	AFR	Gulu, Uganda	1	40	2	CC, CSF	259	98400	79.0	0.203
Yaro et al., 2006	AFR	Bobo-Dioulasso, Burkina Faso	1	24	1	CSF, LA, PCR	264	139795	94.4	0.303

AUTHOR/YEAR OF PUBLICATION	WHO REGION	STUDY SITE	STUDY DESIGN	STUDY DURATION (mo)	STUDY SETTING	DIAGNOSTIC CRITERIA (see legend for abbreviations)	NUMBER OF CASES (CONFIRMED ALL; PRO YEAR)	POPULA-TION DENOMI-NATOR	INCIDENCE (per 100 000, AGE <5 y)	MORTALITY (per 100 000, AGE <5 y)
Traore et al., 2009	AFR	Sotou-boua, Dapaong, Sokode, Togo	1	33	1	CC, CSF, LA, PCR	507	167000	115.6	0.313
Kisakye et al., 2009	AFR	Gulu, Uganda	1	42	2	CC, CSF	259	61198	121.0	NP
Roca et al., 2008	AFR	Manhica, Mozambique	1	48	1	G, BC, LA, CSF	55	11025	124.7	NP
Sigaúque et al., 2008	AFR	Manhica, Mozambique	1	65	1	CC, G, CSF, B	51	7480	125.6	NP
Parent Du Châtelet et al., 2005	AFR	Bobo-Dioulasso, Burkina Faso	1	12	1	CSF, LA, PCR	201	140000	143.6	0.285
Cowgill et al., 2010	AFR	Kilifi, Kenya	1	72	2	CC, CSF, B, LA	343	37614	152.0	NP
O'Dempsey et al., 1996	AFR	Upper River Division, Gambia	1	24	1	B, CSF, LA, BC	31	10000	155.0	NP
Campagne et al., 1999	AFR	Niamey, Niger	2	180	2	CC, G, BC, S, LA, CSF	2671	547743	158.5	0.388
Roca et al., 2006	AFR	Manhica, Mozambique	1	24	1	G, BC, LA, B, CSF	33	9450	174.6	NP
Mwangi et al., 2002	AFR	Kilifi, Kenya	2	84	2	CC, G, CSF, LA	179	12269	208.6	0.317
Adjogble et al., 2007	AFR	Sotou-boua, Dapaong, Sokode, Togo	1	20	2	CC, CSF, LA, PCR	126	27000	280.0	NP

AUTHOR/YEAR OF PUBLICATION	WHO REGION	STUDY SITE	STUDY DESIGN	STUDY DURATION (mo)	STUDY SETTING	DIAGNOSTIC CRITERIA (see legend for abbreviations)	NUMBER OF CASES (CONFIRMED ALL; PRO YEAR)	POPULA-TION DENOMI-NATOR	INCIDENCE (per 100 000, AGE <5 y)	MORTALITY (per 100 000, AGE <5 y)
Roca et al., 2009	AFR	Manhica, Mozambique	1	12	1	G, BC, LA, B, CSF	37	11900	311.0	0.240
Pelkonen et al., 2008	AFR	Luanda, Angola	2	12	2	CC, CSF, LA, PCR	422	NP	NP	0.330
Renner et al., 2007	AFR	Accra, Gana	2	36	2	CC, G, B, CSF	42	NP	NP	0.262
Daza et al., 2006	AFR	Blantyre, Malavi	2	108	2	CSF	1489	NP	NP	NP
Cissé et al., 2010	AFR	Dakar, Senegal	2	57	1	CSF, LA	300	NP	NP	NP
Adams et al., 1993	AMR	USA	2	144	1	CSF, B	643	7600000	8.5	NP
Miranzi et al., 2007	AMR	Brazil	2	192	1	NP	28765	17625610	10.2	0.153
Ribeiro et al., 2007	AMR	Salvador, Brazil	1	24	1	CC, CSF, LA, PCR	66	320000	10.3	NP
Loughlin et al., 1995	AMR	Massachusetts, USA	2	48	2	B, CSF	NP	NP	12.5	NP
Miranzi et al., 2007	AMR	Brazil	2	48	1	NP	10296	16500000	15.6	0.092
Simões LL et al., 2004	AMR	Goiás, Brazil	2	72	1	CSF	308	462673	16.6	NP
Miranzi et al., 2003	AMR	Minas Gerais, Brazil	2	60	1	NP	1868	1787969	20.9	NP
Pérez et al., 2010	AMR	Cuba	2	120	1	CSF, LA, PCR	1429	569000	25.1	0.396

AUTHOR/YEAR OF PUBLICATION	WHO REGION	STUDY SITE	STUDY DESIGN	STUDY DURATION (mo)	STUDY SETTING	DIAGNOSTIC CRITERIA (see legend for abbreviations)	NUMBER OF CASES (CONFIRMED ALL; PRO YEAR)	POPULA-TION DENOMI-NATOR	INCIDENCE (per 100 000, AGE <5 y)	MORTALITY (per 100 000, AGE <5 y)
Gomez et al., 2000	AMR	Santo Domingo, Dominican Republic	1	12	1	CC, CSF, LA	111	329000	33.7	0.207
Ribeiro et al., 2007	AMR	Salvador, Brazil	1	36	1	CC, CSF, LA, PCR	424	320000	44.1	NP
Weiss et al., 2001	AMR	Campinas, Brazil	2	24	1	CC, G, CSF, B, PCR	92	77150	59.6	0.141
Zaidi et al., 2010	EMR	Hyderabad, Karachi, Sindh, Pakistan	1	12	1	CC, CSF, LA	237	2526535	3.1	NP
Shabani et al., 2006	EMR	Kuwait	2	12	2	CC, CSF	16	184000	8.7	0.063
Dash et al., 2008	EMR	Oman	2	72	2	CC, CSF, LA	170	260000	11.0	0.041
Mahmoud et al., 2002	EMR	Al-Ain, UAE	2	120	2	CC, CSF, B	67	19520	34.3	NP
Al-Mazrou et al., 2004 Abstract	EMR	Saudi Arabia	1	24	1	CC, CSF, B	141	171818	41.0	NP
Al Khorasani et al., 2006	EMR	Sa’ada, Jemen	2	24	1	CC, CSF, LA	89	103484	43.0	0.146
Uduman et al., 2000 Abstract	EMR	Abu Dhabi, UAE	1	24	2	CC, CSF, B	18	19520	43.0	NP
Levy et al., 2008	EUR	France	1	84	2	CC, G, CSF, B, LA, PCR	2342	3773500	8.9	0.107
Rossi et al., 2009	EUR	Lazio, Italy	2	60	2	CSF, B	170	230140	14.8	NP

AUTHOR/YEAR OF PUBLICATION	WHO REGION	STUDY SITE	STUDY DESIGN	STUDY DURATION (mo)	STUDY SETTING	DIAGNOSTIC CRITERIA (see legend for abbreviations)	NUMBER OF CASES (CONFIRMED ALL; PRO YEAR)	POPULA-TION DENOMI-NATOR	INCIDENCE (per 100 000, AGE <5 y)	MORTALITY (per 100 000, AGE <5 y)
Kyaw et al., 2002	EUR	Scotland, United Kingdom	2	96	1	CSF, CC, LA	412	322534	16.0	NP
Hudeckova et al., 2010	EUR	Slovakia	2	132	1	CSF, CC, LA	495	276000	16.2	NP
Kojouharova et al., 2003	EUR	Pleven, Plovdiv, Sofia, Stara Zagora, Varna, Bulgaria	2	60	1	CC, G, CSF, LA	137	149431	18.3	NP
Syrogiannopoulos et al., 1995	EUR	Patras, Greece	2	60	2	CC, G, CSF, LA	34	35000	19.4	NP
Tsolia et al., 1998	EUR	Athens, Greece	1	24	2	CSF, B, LA	73	175395	20.8	0.000
Kojouharova et al., 2002	EUR	Pleven, Plovdiv, Sofia, Stara Zagora, Varna, Bugarska	1	30	1	CC, G, CSF, LA	88	138249	25.5	0.200
Dagan et al., 1999	EUR	Israel	1	48	2	CSF, B	588	523615	28.1	NP
Dagan et al., 1994	EUR	Israel	1	36	2	CSF, B	439	492500	29.7	NP
Luca et al., 2004	EUR	Iasi, Constanta, Romania	1	25	2	CC, G, CSF, LA	57	89300	30.6	0.053

AUTHOR/YEAR OF PUBLICATION	WHO REGION	STUDY SITE	STUDY DESIGN	STUDY DURATION (mo)	STUDY SETTING	DIAGNOSTIC CRITERIA (see legend for abbreviations)	NUMBER OF CASES (CONFIRMED ALL; PRO YEAR)	POPULA-TION DENOMI-NATOR	INCIDENCE (per 100 000, AGE <5 y)	MORTALITY (per 100 000, AGE <5 y)
Urwin et al., 1994	EUR	Northeast Thames, United Kingdom	1	36	2	CSF, CC, LA	262	267406	32.6	NP
Kyaw et al., 2002	EUR	Scotland, United Kingdom	2	108	1	CSF, CC, LA	1070	326046	36.6	NP
Skoe et al., 2009	EUR	Sør-Trøndelag, Norway	2	244	2	CSF, B	112	NP (do 16. god)	NP (do 16. god)	0.054
Minz et al., 2008	SEAR	Vellore, Tamil Nadu, India	1	24	1	CC, G, CSF, LA	16	56 153	14.2	NP
Batuwanthudawe et al., 2009	SEAR	Colombo, Sri Lanka	1	27	2	CSF, LA	85	179149	21.0	NP
Shah et al., 2009	SEAR	Kathmandu, Nepal	1	28	2	CC, G, CSF, LA	61	97338	26.8	0.049
Batuwanthudawe et al., 2010	SEAR	Colombo, Sri Lanka	1	12	1	CSF, LA	108	179103	60.3	0.025
Williams et al., 2009	SEAR	Kathmandu, Nepal	1	21	2	CC, G, CSF, LA	38	32500	66.8	NP
Kamiya et al., 1998 (Abstract)	WPR	Japan	2	12	2	NP	74	1411000	5.3	NP
Lee YS et al., 2000	WPR	Singapore	2	72	2	CC, G, CSF, LA	83	223403	6.2	NP
Sakata et al., 2005 (Abstract)	WPR	Hokkaido, Japan	2	60	2	NP	74	234920	6.3	0.048

AUTHOR/YEAR OF PUBLICATION	WHO REGION	STUDY SITE	STUDY DESIGN	STUDY DURATION (mo)	STUDY SETTING	DIAGNOSTIC CRITERIA (see legend for abbreviations)	NUMBER OF CASES (CONFIRMED ALL; PRO YEAR)	POPULA-TION DENOMI-NATOR	INCIDENCE (per 100 000, AGE <5 y)	MORTALITY (per 100 000, AGE <5 y)
Dong BQ et al., 2004 (Abstract)	WPR	Nanning, Guangxi, China	1	26	2	CC, G, CSF	38	140000	12.4	0.184
Kim et al., 2004	WPR	Jeonbuk, South Korea	1	24	1	CC, G, CSF, LA, PCR	30	116894	12.8	0.033
Yang et al., 1996 (Abstract)	WPR	Hefei, China	1	36	2	CC, G, CSF	46	79861	19.2	0.117
Hanna et al., 1991 (Abstract)	WPR	Western provinces, Australia	2	60	2	CSF	270	126000	42.9	0.059
Wilson et al., 2003	WPR	Fiji	2	96	2	CC, G, CSF, LA	394	93993	52.5	NP
Mendsaikhan et al., 2009	WPR	Ulaan Baatar, Mongolia	1	36	2	CC, G, CSF, LA, PCR	111	60047	61.6	0.108
Russel et al., 2003	WPR	Tonga, Polynesia	2	31	2	CC, G, CSF	21	9716	83.4	NP
Dang D. Anh et al., 2006	WPR	Hanoi, Vietnam	1	24	2	CC, G, CSF, LA, PCR	166	94529	88.0	0.048
Russel et al., 2003	WPR	Solomon Islands, Melanesia	2	24	2	CC, G, CSF	63	25703	122.6	NP
Russel et al., 2003	WPR	Samoa, Polynesia	2	36	2	CC, G, CSF	79	17578	149.6	NP
